# Deep Genome-Wide Measurement of Meiotic Gene Conversion Using Tetrad Analysis in *Arabidopsis thaliana*

**DOI:** 10.1371/journal.pgen.1002968

**Published:** 2012-10-04

**Authors:** Yujin Sun, Jonathan H. Ambrose, Brena S. Haughey, Tyler D. Webster, Sarah N. Pierrie, Daniela F. Muñoz, Emily C. Wellman, Shalom Cherian, Scott M. Lewis, Luke E. Berchowitz, Gregory P. Copenhaver

**Affiliations:** 1 Department of Biology and the Carolina Center for Genome Sciences, The University of North Carolina at Chapel Hill, Chapel Hill, North Carolina, United States of America; 2 Curriculum in Genetics and Molecular Biology, The University of North Carolina at Chapel Hill, Chapel Hill, North Carolina, United States of America; 3 Lineberger Comprehensive Cancer Center, The University of North Carolina School of Medicine, Chapel Hill, North Carolina, United States of America; The Pennsylvania State University, United States of America

## Abstract

Gene conversion, the non-reciprocal exchange of genetic information, is one of the potential products of meiotic recombination. It can shape genome structure by acting on repetitive DNA elements, influence allele frequencies at the population level, and is known to be implicated in human disease. But gene conversion is hard to detect directly except in organisms, like fungi, that group their gametes following meiosis. We have developed a novel visual assay that enables us to detect gene conversion events directly in the gametes of the flowering plant *Arabidopsis thaliana*. Using this assay we measured gene conversion events across the genome of more than one million meioses and determined that the genome-wide average frequency is 3.5×10^−4^ conversions per locus per meiosis. We also detected significant locus-to-locus variation in conversion frequency but no intra-locus variation. Significantly, we found one locus on the short arm of chromosome 4 that experienced 3-fold to 6-fold more gene conversions than the other loci tested. Finally, we demonstrated that we could modulate conversion frequency by varying experimental conditions.

## Author Summary

During the production of gametes, most sexually reproducing organisms undergo meiotic recombination. The most familiar form of meiotic recombination is crossing-over, which results in the reciprocal exchange of DNA between parental chromosomes and is important for chromosome segregation as well as generating new allelic combinations in progeny. The same molecular mechanisms that facilitate crossing-over can also enable the non-reciprocal exchange of genetic information between chromosomes in the process called gene conversion. Understanding gene conversion is important because it influences allele frequencies and has been implicated in human diseases. Unfortunately, it has been difficult until now to measure directly except in organisms, like fungi, that group their gametes after meiosis. In this study we have developed a novel assay system that enables us to measure gene conversion directly in the model multi-cellular eukaryote *A. thaliana* (a flowering plant). Using this assay system we measured gene conversion frequencies across the Arabidopsis genome in more than 1 million meioses and also demonstrated that we can manipulate those frequencies by varying experimental conditions.

## Introduction

Gene conversion is the replacement of DNA sequence at one locus using a second locus as a template. It can occur between alleles or between non-allelic sequences that share homology and during mitotic or meiotic recombination, but unlike crossovers it does not result in the reciprocal exchange of DNA [1], . Research interest in gene conversion is motivated by its roles in human pathogenesis, genome dynamics and evolution as well as its usefulness in evaluating mechanistic models of recombination [3]. Despite its importance, gene conversion has been difficult to study except in organisms, like many fungi, that retain their meiotic products in distinct groupings. We have used a novel gene conversion reporter system in the flowering plant *Arabidopsis thaliana* to measure allelic gene conversion frequencies in over a million meioses.

**Figure 1:**
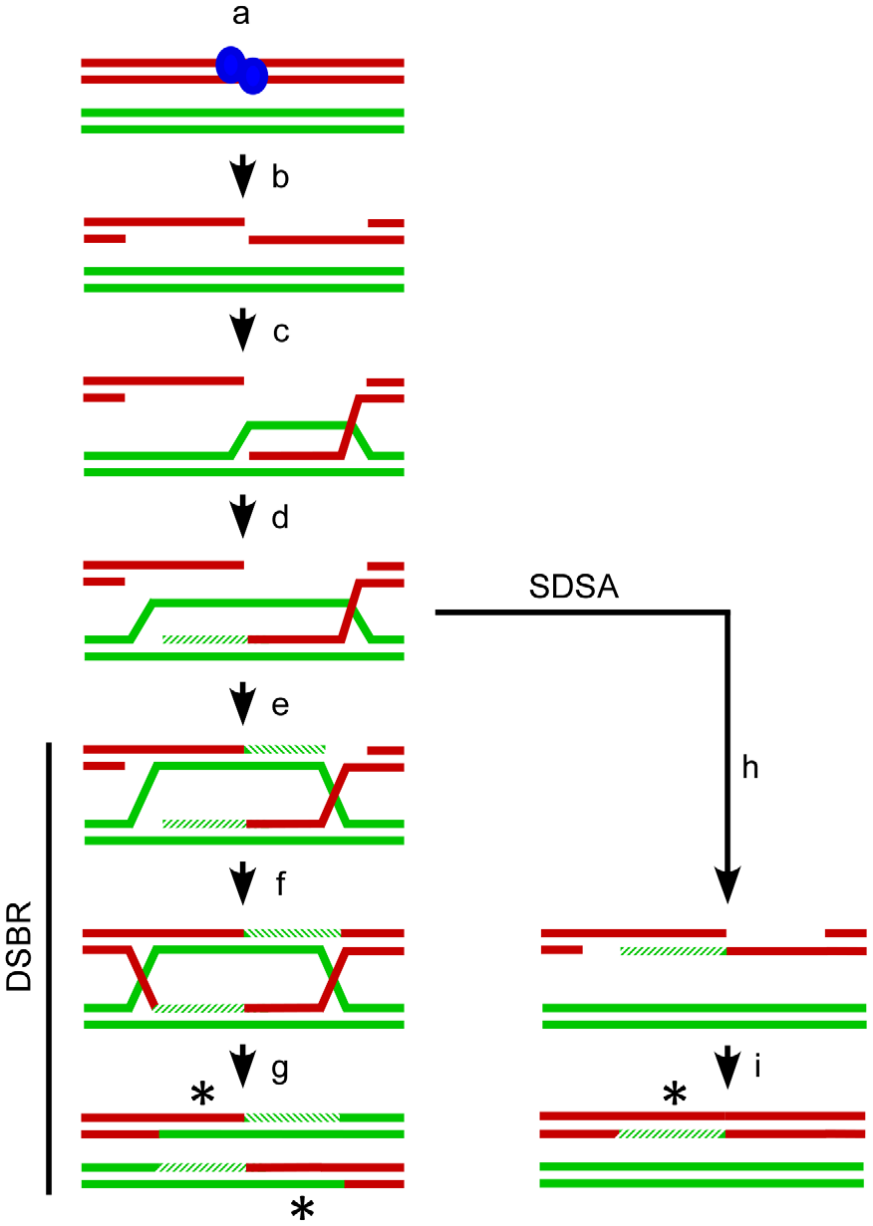
Meiotic recombination models. The Watson and Crick strands (red and green lines) for two of the four chromatids present at meiosis are shown. Recombination is (a) initiated by SPO11 (blue ovals) catalyzed breaks in one chromatid followed by (b) release of SPO11 and further resection to generate single-stranded 3′ tails. One tail (c) invades a non-sister chromatid to form a D-loop and (d) the invading strand can be extended by DNA polymerase (hatched lines). The Double Strand Break Repair (DSBR, left) pathway proceeds with (e) the D-loop capturing the second 3′ end which is also extended by DNA polymerase. Ligation of the available ends (f) generates a double Holliday Junction which is can be resolved (g) as a crossover with associated regions of heteroduplex DNA (asterisks). Alternatively in Synthesis Dependent Strand Annealing (SDSA, right) the invading strand can dissociate from the homologous chromatid prior to second end capture (h) and re-anneal to the 3′ end on the other side of the break. Gap synthesis and ligation (i) will produce a non-crossover with associated heteroduplex DNA (asterisk).

Meiotic recombination is initiated by the formation of DNA double-strand breaks (DSBs) catalyzed by the phylogenetically conserved protein SPO11 (Figure 1) [4]. The canonical model for recombination, called Double Strand Break Repair (DSBR), begins with release of SPO11 from either side of the break leaving 3′ overhangs that are further resected to form 3′ tails [5]. A 3′ tail can invade a homologous chromosome by annealing to its complement and displacing the other strand to form a D-loop. This annealing process results in heteroduplex DNA [6]. As a result, any sequence polymorphisms between the homologous chromosomes will yield mismatches in the heteroduplexed region. DNA polymerase can extend the invading 3′ end using the homologous chromosome as a template thereby expanding the D-loop until it can capture the second 3′ end of the break. Annealing of the D-loop to the second 3′ end also produces heteroduplex DNA, and polymorphisms will again yield mismatches. DNA synthesis primed by the second 3′ end, followed by ligation results in the recombination intermediate known as a double Holliday Junction (dHJ). Theoretically, dHJs can be enzymatically resolved to produce either crossovers (COs) or non-crossovers (NCOs). However, biochemical studies in *Saccharomyces cerevisiae* examining the temporal sequence of DSBs, dHJs, COs and NCOs suggest that dHJs are preferentially resolved to produce COs [7]. While the origin of NCOs in plants has yet to be determined, in *S. cerevisiae* they are thought to be produced primarily by an alternative pathway called Synthesis Dependent Strand Annealing (SDSA) [8]. SDSA diverges from the DSBR pathway at the point of second-end capture. The D-loop does not capture the second 3′ end. Instead, the invading strand dissociates from the homologous chromosome after extension by DNA polymerase and anneals to the second end. Gap filling and ligation can then produce a NCO. Because extension of the invading end uses a homologous chromosome as a template, re-annealing to the second end will also produce mismatches if polymorphisms are present.

The DSBR and SDSA pathways both generate heteroduplex DNA making DNA mismatches possible. The Mismatch Repair (MMR) system can recognize and mend such lesions [9]. If MMR restores the original allelic state, the expected Mendelian 2∶2 ratio of alleles is maintained at the locus. The alternative is for MMR to repair the mismatch in favor of the homologous allele, resulting in gene conversion and a tell-tale non-Mendelian 3∶1 ratio of alleles. Absent any other considerations, MMR should produce conversions and restorations in a 50∶50 ratio. There is evidence, however, for biased gene conversion that favors alleles on the chromatid that did not experience the initiating DSB, as well as bias for the generation of G/C base pairs [10]–[12].

The term “gene conversion” was coined by Hans Winkler, who saw it as the basis for all recombination [13]. Gene conversion as an event resulting in the non-reciprocal exchange of genetic information was first observed by H. Zickler in the octads of the ascomycete *Bombardia lunata* [14]. In *S. cerevisiae*, where it has been studied most comprehensively, gene conversion converts up to 1% of the genome (92–320 kb from COs and 62–148 kb from NCOs) in each meiosis [15]. Gene conversion is more difficult to measure directly in multicellular eukaryotes, including humans and plants, because their gametes do not typically remain grouped after meiosis, so the classic 3∶1 signature cannot be observed. Instead, gene conversion is assumed to have occurred in these systems when a polymorphic marker switches parental type, but closely spaced flanking markers don't experience exchange. Similar analyses of haplotypes at the population level enable estimation of historic gene conversion frequencies [16]. Formally, these indirect observations could also result from closely spaced double crossovers, but crossover interference is assumed to limit such events making their influence negligible [17]. Despite these detection difficulties, gene conversion has been implicated in at least 18 human diseases and is important in shaping linkage disequilibrium in the genomes of most eukaryotes [3].

Gene conversion in plants has been measured primarily at specific loci or limited regions. For example, Shi et al. used indirect measurement techniques similar to those described above and estimated that there are between 1×10^−5^ and 3.7×10^−5^ gene conversions per marker per generation in the functional centromeres of maize [18]. This observation is a particularly interesting because in maize, like most eukaryotes, COs are thought to be essentially absent from centromeres [19], [20]. Thus, the detection of gene conversion events implies that these regions still experience DSBs that are presumably repaired by NCOs or by sister repair. More recently, Lu et al. sequenced the products of two meioses from an F_1_ hybrid between the Landsberg and Columbia ecotypes of Arabidopsis [21]. They observed 18 COs, six of which were associated with conversion tracts. They also observed 4 NCO gene conversions for a total of 10 conversions in two meioses. This analysis is powerful in that it has nucleotide resolution and is limited only by the density of polymorphisms between the two parental ecotypes and the number of tetrads that can be feasibly sequenced. Our analysis takes a different approach – we measured GC at a limited number of loci across the genome but examined over a million meioses.

**Figure 2:**
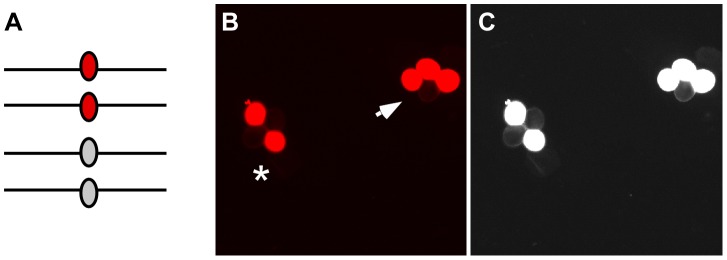
Gene conversion test loci. (A) After pre-meiotic DNA synthesis, meiocytes in plants that are heterozygous for fluorescent and non-fluorescent alleles of the FTL transgene cassettes will have two copies of each allele. (B) Following meiosis those alleles will segregate in pollen tetrads. If no gene conversion occurs at the test locus the fluorescent signal will segregate in a 2∶2 ratio (asterisk). In contrast, gene conversion will result in a 3∶1 segregation ratio (arrow). (C) A monochrome image with the exposure and contrast globally increased using Photoshop enables easier visualization of the non-fluorescent pollen grains in the tetrads.

We developed a novel visual assay that enables us to measure COs, NCOs and GCs directly in the gametes of Arabidopsis [22]. Previously, we had generated a collection of transgenic plants with transgenes encoding fluorescent proteins expressed by a pollen-specific, post-meiotic promoter called *LAT52* in a *quartet1–2* (*qrt1–2*) mutant background that produces persistent pollen tetrads (we call these plants Fluorescent Tagged Lines or FTLs) [23]–[26]. The segregation of the fluorescent proteins in the pollen tetrads is a direct reflection of the segregation of the transgenes that encode them. As a result, when transgenes encoding fluorescent proteins of different colors are linked on the same chromosome, their expression patterns will differ in pollen tetrads if a CO occurs in the interval between them [27]. To detect GCs, we modified the FTL system by generating non-fluorescent mutant alleles of our existing transgenic lines. Pollen tetrads from plants that have heterozygous fluorescent and non-fluorescent alleles at a transgene locus will typically segregate fluorescence in a 2∶2 ratio (Figure 2). If GC occurs at the test locus, however, a non-Mendelian 3∶1 ratio is observed. This system has two significant advantages. Large numbers of tetrads (meioses) can be scored by visual analysis in a relatively short time, and the analysis can occur in an otherwise isogenic background that limits the influence of sequence heterogeneities between polymorphic parents [28], [29]. Using this system, we scored the frequency of gene conversion at 7 test loci distributed among the 5 Arabidopsis chromosomes in over a million tetrads to provide a genome-wide estimate of GC frequency and locus to locus variation. We also measured the ratio of CO∶NCO associated GCs as well as how environmental queues and developmental status influence GC frequencies.

## Results

### Generating GC Test Loci

To measure meiotic COs in Arabidopsis, we had previously generated a collection of *qrt1–2^−/−^* plants with transgenes encoding fluorescent proteins (eYFP, DsRed2 and AmCyan) expressed under the control of the post-meiotic, pollen-specific promoter *LAT52* [23]. To modify this system so that it could also be used to measure gene conversion, we created non-fluorescent mutant alleles for a subset of the collection, using ethyl methanesulfonate (EMS) mutagenesis. The *Agrobacterium tumefaciens* transformation method used to create the original FTL lines is known to sometimes insert tandem copies of transgene cassettes [30]. Since tandem transgene copies would reduce the efficiency of our EMS mutagenesis, we first screened our FTL lines by using PCR to amplify the whole transgene cassette from primers in the flanking genomic DNA, and by using ligation mediated suppression PCR to detect which lines carried single-copy transgenes. The FTL lines that passed the PCR screen were verified by hybridizing Southern blots of FTL genomic DNA digested with EcoRI (single recognition site within the transgene cassette) with a probe corresponding to the *LAT52* promoter. Of the 66 FTL lines screened, we identified 10 with single copy transgenes. It should be noted that Agrobacterium mediated transgene insertion is nonrandom with respect to gene density and therefore the distribution of our test loci may be similarly nonrandom [31], [32].

Seed from FTLs homozygous for single copy transgenes were subjected to EMS mutagenesis. Pollen tetrads from the M_1_ plants were observed using epi-fluorescence microscopy to identify individuals expressing a 2∶2 fluorescent:non-fluorescent phenotype. Normally, plants homozygous for the fluorescent transgene will express a 4∶0 fluorescent pollen phenotype; hence, a 2∶2 phenotype indicates that one of the fluorescent protein alleles has suffered a mutation. We identified a total of 17 non-fluorescent alleles in 7 of the 10 FTLs on which we attempted mutagenesis (Table 1, Figure S1). At each locus the non-fluorescent alleles (with the exception of 2 alleles which were not used further in the study) were sequenced and the non-fluorescent FTL (NFTL) was backcrossed three or four times to the parental FTL line to eliminate background mutations. Of the 15 alleles sequenced, 10 were G→A changes and 5 were C→T changes in the coding strand, consistent with a strong bias for G/C→A/T transitions by EMS in Arabidopsis and other organisms (Table 1) [33].

**Table 1:** Non-fluorescent tagged line (NFTL) alleles.

NFTL allele	Chrm.	Transgene Nucleotide Position	Fluor	Mutation	AA Change
**567-GC1**	1	3905441	yfp	G95A	G32D
**567-GC2**	1			*	*
**567-GC3**	1			*	*
**3282-GC1**	1	25652977	amcyan	G118A	G40R
**3282-GC2**	1			C224T	T75I
**3282-GC3**	1			G383A	G128E
**3282-GC4**	1			G118A	G40R
**3411-GC1**	2	18957093	yfp	G203A	G68D
**1369-GC1**	3	6472617	dsred	G308A	G103D
**1369-GC2**	3			G428A	W143X
**424-GC1**	4	1365848	yfp	G174A	W58X
**424-GC2**	4			C188T	T63I
**424-GC3**	4			C617T	S206F
**424-GC4**	4			C332T	A11V
**1273-GC1**	5	18164269	dsred	G92A	G31D
**1273-GC2**	5			C223T	H75Y
**1659-GC1**	5	23080567	yfp	G383A	G128D

* Alleles not sequenced or used in this study.

### Genome-Wide Measurement of Gene Conversion Frequencies

To determine how frequently gene conversion occurs in the Arabidopsis genome, we used epi-fluorescence microscopy to examine pollen tetrads from plants heterozygous for the fluorescent and non-fluorescent alleles at one of each of the seven test loci described above. 1,054,024 tetrads were scored (an average of 150,574 per locus), and 186 tetrads with a 3∶1 segregation pattern were observed (Table 2). We also observed tetrads with a 1∶3 segregation pattern, but these were not included in our gene conversion counts since non-fluorescence could be attributed to pollen development defects or other causes. To account for both classes of conversion, we doubled the number of 3∶1 tetrads in all of our calculations. With this adjustment factor, the genome-wide average is 3.5×10^−4^ conversions per locus per meiosis (or 1 conversion per locus in every 2,833 meioses).

**Table 2:** Genome-wide conversion frequencies.

NFTL allele	Chrm.	GC	tetrads	raw f(GC_obs_)	adjusted f(GC_obs_)*	GC per meioses
**567-GC1**	1	17	148886	1.14E-04	2.28E-04	4379
**3282-GC1**	1	17	150910	1.13E-04	2.25E-04	4439
**3411-GC1**	2	30	150706	1.99E-04	3.98E-04	2512
**1369-GC1**	3	16	155280	1.03E-04	2.06E-04	4853
**424-GC1**	4	79	147848	5.34E-04	1.07E-03	936
**1273-GC1**	5	14	150429	9.31E-05	1.86E-04	5372
**1659-GC1**	5	13	149965	8.67E-05	1.73E-04	5768
**total**		186	1054024	1.76E-04	3.53E-04	2833

* Adjusted frequencies double the number of observed 3∶1 tetrads to account for 1∶3 tetrads.

A 3∶1 segregation pattern could also be obtained if one of the non-fluorescent alleles experienced a mutation that restored fluorescence (a reversion). To control for this possibility, we scored 45,000 pollen tetrads from a plant that was homozygous for the NFTL 3282-GC1 allele and observed only 0∶4 tetrads (plant genotypes were confirmed using allele-specific PCR). Since no reversion events were observed, the reversion frequency under our experimental conditions is significantly lower than the GC frequency observed (P = 0.008998). Consistent with these findings, mitotic reversion rates at transgene loci in Arabidopsis have been independently measured between 10^−7^ to 10^−8^ [34]. Another source of false-positives is the mechanical disruption and subsequent random re-association of pollen tetrads to yield a grouping of pollen grains that are not meiotically related. To control for this possibility we scored 45,000 tetrads from a plant that was hemizygous for the FTL 567 locus and observed no 3∶1 tetrads. Since no spurious 3∶1 tetrads were observed the frequency of this type of physical re-association is significantly lower than the observed GC frequency (P = 0.008998). We conclude that most, if not all, the events we observed were due to gene conversion rather than reversion or false-positives.

### Inter- and Intra-Locus Variation in Gene Conversion

Gene conversion frequencies might be expected to vary depending on DSB distribution, CO/NCO balance, bias in restoration versus conversion by MMR, or through the indirect effects of sequence context or epigenetic influences. Alternatively, since gene conversion can alter the genome, there might be regulatory mechanisms constraining its action. To determine if there is locus to locus variation in gene conversion frequency in Arabidopsis, we looked for statistical differences in all pairwise combinations of our seven test loci (Table 3). NFTL 424-GC1 experienced significantly more gene conversion than all other test loci (P<2×10^−11^ to 2×10^−15^). NFTL 3411-GC1 also had a significantly higher gene conversion frequency when compared to 4 of the 6 other loci (P<1×10^−6^ to 0.04). NFTL 1659-GC1 and 1273 GC-1 experienced the lowest frequency of gene conversions and were significantly lower than the genomic average (P<0.02 and 0.01 respectively), as was NFTL 1369 GC-1 (P<0.04). We conclude that there is significant locus-to-locus variation in gene conversion in Arabidopsis.

**Table 3:** Variation of gene conversion frequencies (P values).

	567-GC1	3282-GC1	3411-GC1	1369-GC1	1369-GC2	424-GC1	1273-GC1	1659-GC1	total
**567-GC1**	-	0.97	0.06	0.77	0.83	3E-11*	0.57	0.45	0.11
**3282-GC1**		-	0.06	0.80	0.86	2E-11*	0.60	0.48	0.10
**3411-GC1**			-	0.03*	0.04*	1E-6*	0.02*	0.01*	0.39
**1369-GC1**				-	0.94	3E-12*	0.78	0.64	0.04*
**1369-GC2**					-	8E-12*	0.72	0.59	0.06
**424-GC1**						-	8E-13*	3E-13*	2E-15*
**1273-GC1**							-	0.85	0.02*
**1659-GC1**								-	0.01*
**total**									-

* Indicates significance at P<0.05.

Given the locus-to-locus variation observed, we asked whether there was intra-locus variation as well by comparing the gene conversion frequency of two different alleles, NFTL 1369-GC1 and 1369-GC2 (the SNPs in each allele are separated by 120 bp). After counting 155,280 and 150,916 tetrads respectively, we observed 32 (adjusted) conversions for each allele indicating - at least for this locus - a lack of intra-locus variation (P<0.94, Table 3).

**Figure 3:**
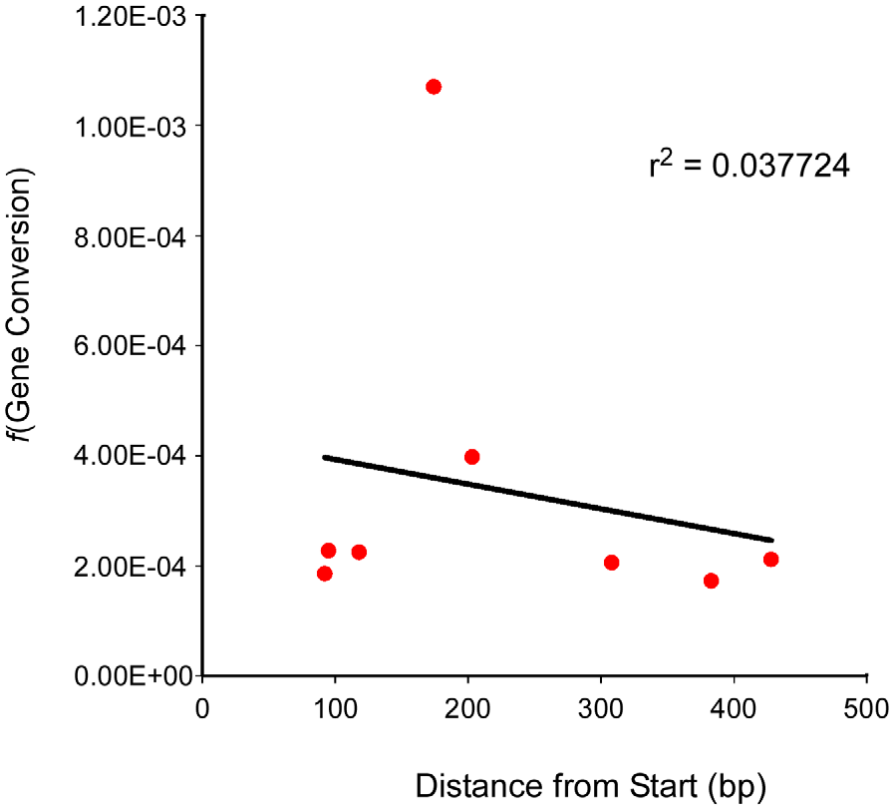
Test locus SNP position and conversion frequency lack correlation. The frequency of gene conversion (Y-axis) at each test locus (and for one locus, two different alleles) was plotted against the position of the polymorphic marker (SNP) at that locus (X-axis). A linear regression for the scatter plot was generated as well as an r^2^ value.

Evidence from *S. cerevisiae* has demonstrated that the frequency of gene conversion can exhibit polarity, typically (though not always) with a higher level at the 5′ end of genes [35], [36]. This polarity is thought to result from either a gradient in DSB formation, with a preference for promoter regions, or modulation of the direction of mismatch repair (conversion versus restoration) [37]–[39]. To see if we could detect a similar polarity for gene conversion in Arabidopsis we plotted the position of the mutation at each of our test loci relative to the transcriptional start site (Table 1) against the frequency of gene conversion observed at each locus (Table 2, Figure 3). The linear regression of position of the polymorphism on conversion frequency was not significant (r^2^ = 0.038, P = 0.645). The result of this limited analysis (seven loci) supports the prior suggestion that Arabidopsis lacks strong intra-locus variation in gene conversion frequency.

### The Balance between CO– and NCO–Associated Gene Conversions

**Figure 4:**
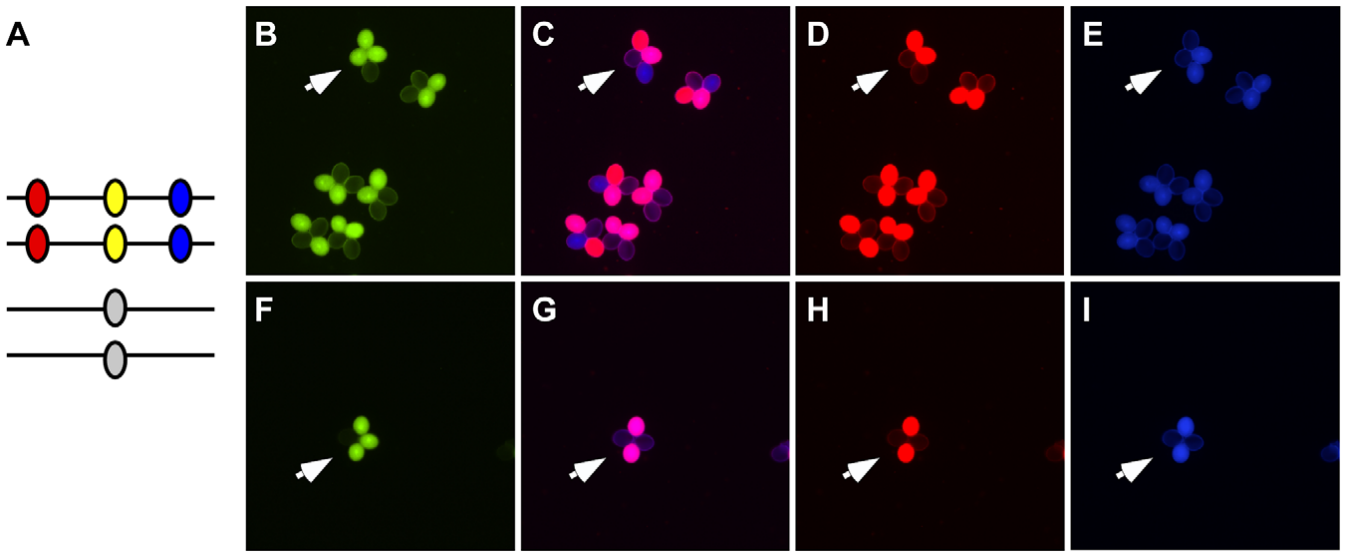
Assigning conversions to COs or NCOs. (A) A heterozygote with DsRed (red oval), eYFP (yellow oval) and AmCyan (blue oval) transgenes on one homolog and a non-fluorescent allele of eYFP on the other homolog was used to detect gene conversion events (arrows in B and F). These gene conversions could be categorized as being associated with crossover (C–E) or non-crossovers (G–I) by determining whether the flanking DsRed and AmCyan markers experienced exchange.

As described above, COs produced by DSBR and NCOs produced by SDSA can both be accompanied by GC. To determine what proportion of the conversions we observed can be attributed to either pathway, we constructed a combination of markers that enables us to monitor both conversions and COs simultaneously. We flanked our NFTL 1659-GC1 test locus on chromosome 5 with FTL 1273 (DsRed2) and FTL 993 (AmCyan) on either side (Figure 4). When conversions at NFTL 1659-GC1 were observed, they could be categorized as CO-GC or NCO-GC by scoring the segregation of the flanking markers. We scored 149,965 tetrads and observed 13 conversions. 11 were associated with COs and 1 was associated with a NCO (the flanking AmCyan signal in the remaining tetrad was too weak to score unambiguously). These results demonstrate the utility of our system for measuring the balance between CO and NCO associated GCs, however measurements at similar triple-color test intervals across the genome will be necessary before broad conclusions can be drawn.

### Modulating Gene Conversion Frequencies

Using our FTL system, we had previously demonstrated that CO frequencies can be significantly elevated in Arabidopsis either by growth at high temperatures or by sampling meioses from 2° or 3° axes (branches) rather than the 1° shoot (i.e. different developmental contexts) [23]. To determine whether gene conversion is also influenced by these environmental and developmental queues, we scored tetrads from plants that were heterozygous for the NFTL 424-GC1 marker under the same experimental conditions. Surprisingly, unlike their influence on CO frequency the treatments showed divergent effects on gene conversion. The elevated temperature treatment increased conversion frequencies significantly. Control plants grown at 20°C experienced 1 conversion in every 936 meioses (Table 2; n = 147,848 tetrads), while plants grown at 28°C had 1 conversion in every 321 meioses (P<8×10^−4^; n = 9,624 tetrads). By contrast, tetrads (n = 160,581) collected from branches showed no significant change in conversions with one event per 1,147 meioses (P<0.21) compared to controls.

## Discussion

To understand the molecular mechanisms that facilitate and regulate meiotic recombination, a useful experimental system permits easy and rapid analysis of COs, NCOs and GCs. We had previously used our FTL system to assay crossing-over, crossover interference and mutants influencing those processes [23], [40]. Here we've adapted the FTL system to enable measurement of GC frequencies and detection of NCOs, and we've established a baseline description of the meiotic gene conversion landscape in Arabidopsis.

A limitation of the current version of this system is that it detects the conversion of a single SNP at the test locus. As a result, direct measurement of the length of DNA that is converted in a given event (i.e. the “conversion tract” length) is not possible. Nonetheless, our data can provide a tentative tract length estimate. The sequenced portion of the Arabidopsis genome comprises 119,146,348 nucleotides. The five centromeres and two ribosomal RNA gene arrays have not been sequenced and may contain an additional 15 Mb and 7 Mb of DNA respectively for an upper genome estimate of 141,146,348 bases (GenBank Assembly ID: GCA 000001735.1) [41]–[44]. Unlike *S. cerevisiae*, the number of DSBs per meiosis has not been measured directly in Arabidopsis, but several studies using counts of RAD51 foci at mid-prophase as a proxy for DSBs suggest that there are likely between 120 and 222 breaks [45]–[47]. Assuming each of those breaks is repaired by either the DSBR or the SDSA pathway, associated conversion tracts should be possible for all of them (Figure 1). If MMR restores 50% of those events to their original allelic state, we would expect 60–111 conversions across the genome. Dividing the high and low estimates of the genome size by the low and high estimates of the number of conversions yields an expected frequency of 4.25×10^−7^ to 9.32×10^−7^ gene conversion events per nucleotide per meiosis. But this is several orders of magnitude lower than our observed frequency of 3.5×10^−4^. The observed and expected frequencies can be reconciled if a tract length of 379–830 bp (average 605 bp) is assumed. This estimate is tenuous since it rests on several assumptions (numbers of DSBs, frequency of homolog versus sister repair, efficiency and directionality of MMR during meiosis), but it corresponds nicely to the median midpoint length of 558 bp that Lu et al. provided, based on their analysis of a limited number of conversions in Arabidopsis [21]. It may be possible in the future to modify our system further by incorporating additional SNPs at the test loci to enable direct measurement of tract lengths.

As outlined above, the roughly 120–222 DSBs (as estimated by RAD51 foci) that occur in each Arabidopsis meiosis are thought to be repaired by either the DSBR or the SDSA pathways. Only a small fraction of these are repaired as COs (DSBR pathway): numerous studies have demonstrated that Arabidopsis experiences ∼9 COs per meiosis [21], ; so the remaining 111–213 breaks should be repaired as NCOs (SDSA pathway). This implies that the CO∶NCO balance in Arabidopsis should be ∼1∶12 to 1∶20 - in stark contrast to our observed ratio of 11 COs for each NCO. As described earlier, Lu et al. also observed more COs than NCOs after sequencing the equivalent of two tetrads.

There are several ways to explain this. Most trivially, the particular locus we measured could be anomalous. A more interesting possibility is that most meiotic DSBs in Arabidopsis may be repaired using a sister chromatid rather than a non-sister chromatid. Sturtevant's discovery of unequal crossing over at the *Bar* locus in Drosophila initiated the idea that there is a strong bias for meiotic inter-homolog rather than sister exchange [50]. The possibility of inter-homolog bias was bolstered by experiments in *S. cerevisiae* and Drosophila measuring recombination between structurally heterozygous chromosomes (ring/rod heterozygotes) [51], [52]. These findings are consistent with more recent experiments showing that the majority of the ∼160 DSBs/meiosis in yeast are repaired as inter-homolg COs or NCOs (∼137 total/meiosis) [53].

However, similar experiments looking at recombination in ring/rod heterozygotes in maize and *Antirrhinum majus* (snapdragons) suggest that meiotic sister chromatid exchange may occur more frequently in plants [54], [55]. Indeed, even in yeast where inter-homolog bias is thought to be robust, when DSBs occur in regions lacking a homologous locus the breaks are efficiently repaired from the sister [56]. Alternatively, most DSBs in plants could be repaired as inter-homolog COs or NCOs but MMR in the SDSA pathway may be disproportionately biased in favor of restoration rather than conversion, resulting in fewer detectable NCOs. Another possibility is that conversion tracts associated with COs may be longer than those associated with NCOs and are detected at a given test locus more frequently. This idea is supported by data from *S. cerevisiae*, which has an average conversion tract length of 2.0 kb and 1.8 kb for COs and NCOs respectively (P<0.0001) [15].

The balance between inter-homolog versus sister exchange or bias in restoration versus conversion by the MMR machinery may also contribute to our results when trying to modulate gene conversion frequency. An increase in both COs and conversions may indicate that more DSBs are formed in Arabidopsis at elevated temperatures. Alternatively, DSBs may be held constant, but under normal growth conditions some breaks are repaired from sister chromatids while under elevated temperatures sister-repair is directed instead to homologous repair and manifests as COs and NCOs associated with conversions. To differentiate these possibilities, it will be necessary to use mutant analysis.

We've built on our previous visual assay for COs in Arabidopsis and expanded it so that it can now measure both GCs and NCOs as well. Using this system, we've characterized the baseline gene conversion landscape during meiosis to serve as a useful reference point for future analyses. We've also demonstrated that the system can be used to detected experimentally induced changes in gene conversion frequencies. This provides a proof of principle that will enable the system to be used to investigate which proteins mediate and regulate meiotic recombination in plants.

## Materials and Methods

### Plants and Growth Conditions

All FTL lines have been described previously and are available from G. P. Copenhaver upon request, as are all the lines generated in this study [23]. Seeds were sown on a pre-wetted 5∶1 mix of Metro-Mix 360 soil (Sun Gro Horticulture, http://www.sungro.com/) and Horticultural Perlite (Carolina Perlite Company, Inc., Gold Hill, NC, USA) and stratified for 3–4 days at 4°C. Plants were grown under long-day conditions (18 h day, 6 h night) at 20°C unless otherwise noted.

### DNA Extraction and Analysis

Genomic DNA was extracted from ∼100 mg of fresh cauline leaves using Plant DNAzol (Live Technologies, www.invitrogen.com) per the manufacturer's instructions. To identify single-copy FTL lines, PCR primers corresponding to genomic sequences flanking the transgene (P1 and P2 – specific to each FTL line) were used in combination with primers corresponding to the left or right T-DNA sequence (L1 or R1) to amplify genomic-T-DNA junctions from FTL homozygotes (see Table S1 for primer sequences). PCR amplification using P1/P2/L1 or R1 will yield a single PCR product from single-copy FTL lines. The same primers (P1, P2, L1 or P1, P2, R1) amplify only genomic sequence from control wild-type plants. Amplification of whole transgene cassettes from putative single-copy FTL lines was achieved using LongAmp DNA polymerase (New England BioLabs, www.neb.com) with primers P1 and P2. Allele-specific genotyping was conducted using a P1 and L1 or R1, or P2 and L1 or R1. Ligation mediate suppression (LMS) PCR, as described previously, was used to verify single-copy transgene cassettes in FTL lines [23], [57].

Single copy transgenes were verified using Southern blotting as described by Forsbach et al. [58]. Genomic DNA was digested with EcoRI, which targets a single site within the transgene cassette, and separated by electrophoresis using an 0.8% agarose gel. Gels were denatured with 0.4 M NaOH and transferred to Zeta-Probe GT nylon membranes (BioRad, www.bio-rad.com). Probes were generated by PCR using primers for the *LAT52* promoter (see Table S1 for primers), labeled with ^32^P dATP or dCTP using the DECAprime II labeling kit (Life Technologies), and purified with NucAway spin columns (Life Technologies). Blots were pre-hybridized in 0.25 M Na_3_PO_4_ (pH 7.2), 7% SDS for 1 hour at 65°C and then hybridized with the probe in 15 ml of the same solution overnight at 65°C with agitation. Hybridized blots were washed twice in 20 mM Na_3_PO_4_ (pH 7.2), 5% SDS for 30 minutes at 65°C followed by a 20 mM Na_3_PO_4_ (pH 7.2), 1% SDS wash for 30 minutes at 65°C. Hybridization patterns were visualized using a phosphorimager.

### Mutagenesis

120 mg of seed were incubated with gentle agitation at room temperature for 16 hours in 45 ml ddH_2_O with 0.27% ethylmethane sulfonate (EMS). Mutagenized seed were rinsed twice with 45 ml water for 4 hours followed by 9 additional 45 ml rapid rinses. Rinsed seed were suspended in 45 ml of 0.05% agarose and incubated at 4°C for 3 days. The cold treated seeds were transferred to 100 ml of fresh 0.05% agarose solution and planted on soil.

### Microscopy

The segregation of fluorescent marker protein expression in pollen tetrads was measured with either a Nikon (www.nikon.com) E1000 or Eclipse 80i epifluorescence microscope equipped with a Nikon Intensilight C-HGFI light source and filters from Chroma Technology (www.chroma.com). To collect pollen tetrads, flowers were dipped in a 10 µl drop of PGM media (34% sucrose, 4 mM CaCl_2_, 3.25 mM boric acid, 0.1% Triton-X-100, pH 7.5) on a glass microscope slide and covered with a glass coverslip. Photographs were taken with either a Nikon Coolpix5000 digital camera or a Nikon Digital Sight DS-Qi1MC cooled CCD camera. Figures were prepared using Adobe Photoshop CS2 (www.photoshop.com) and Canvas X (www.acdsee.com).

### Statistical Analysis

To test whether the frequency of gene conversion events is independent of genomic position, experimental condition, mutation frequency, or false-positives from physical re-association 2×2 contingencies tables were constructed for each possible pair of test loci (or experimental and control conditions) and the G-Test of Independence was used to generate a P-value with the tools provided at the online version of the Handbook of Biological Statistics (http://udel.edu/~mcdonald/statgtestind.html) [59]. To test for a correlation between the position of the SNP within the test locus (relative to the transcriptional start site) and gene conversion frequency tools from the same site were used to generate a regression line, calculate a correlation coefficient (r^2^) and a P-value.

## Supporting Information

Figure S1 Map of the GC test loci. Each FTL (top colored circles) and NFTL (bottom black circles) allele is displayed on the 5 Arabidopsis chromosomes (green bars) with a scale bar for reference (horizontal black bar). The position of the *QUARTET1* (*QRT1*) locus is also shown.
(TIF)

Table S1 Primers used in this study.
(DOCX)
