# Supplementary figures and images for: Deep Genome-Wide Measurement of Meiotic Gene Conversion Using Tetrad Analysis in Arabidopsis thaliana
Source: PLoS Genet. 2012 Oct 4;8(10):e1002968. doi: 10.1371/journal.pgen.1002968 (PMC3464199; doi:10.1371/journal.pgen.1002968)

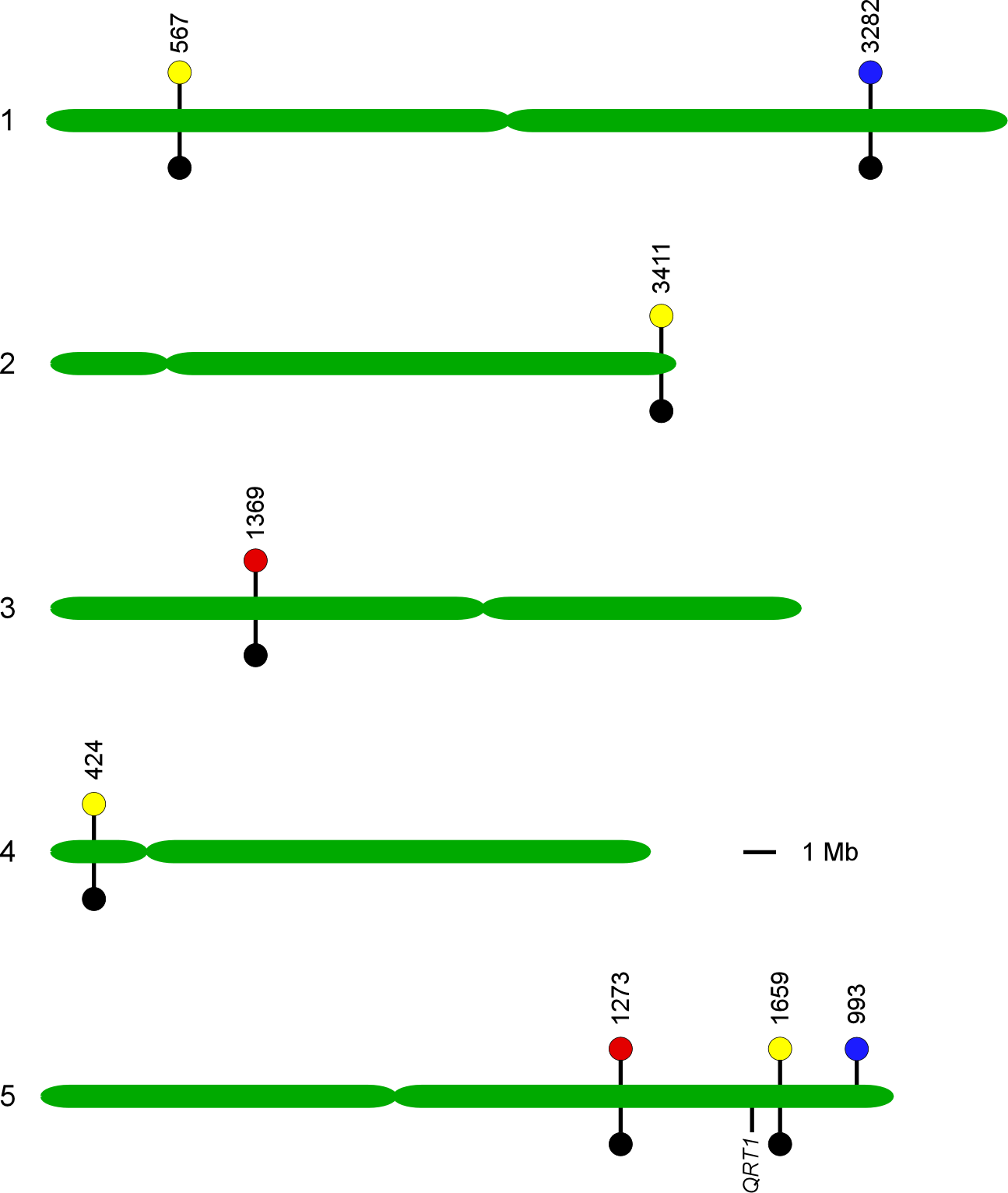

Supplement: Figure S1 — Map of the GC test loci. Each FTL (top colored circles) and NFTL (bottom black circles) allele is displayed on the 5 Arabidopsis chromosomes (green bars) with a scale bar for reference (horizontal black bar). The position of the QUARTET1 (QRT1) locus is also shown. (TIF) [file pgen.1002968.s001.tif]
