# Supplementary material for: Deep Genome-Wide Measurement of Meiotic Gene Conversion Using Tetrad Analysis in Arabidopsis thaliana
Source: PLoS Genet. 2012 Oct 4;8(10):e1002968. doi: 10.1371/journal.pgen.1002968 (PMC3464199; doi:10.1371/journal.pgen.1002968)
Supplement: Table S1 — Primers used in this study. (DOCX) [file pgen.1002968.s002.docx]

Table S1. Primers used in this study

| Primer | Sequence | Notes |
| --- | --- | --- |
| FTL1019-P1 | ACCATCATCATACCCACCGCTCTA |  |
| FTL1019-P2 | AAATCGGAGGAGTGAGCACTGAAG |  |
| FTL1046-P1 | TCCACATCCTCCTCCTCACAGC |  |
| FTL1046-P2 | TGACCAAACGAAGAACCCTGAAG |  |
| FTL1056-P1 | ATCTTTCAATCCCTCGGTGCTAC |  |
| FTL1056-P2 | AACCTCACCGATTTGGCCTTGTC |  |
| FTL1065-P1 | ACTTGAGGCGAAGCGTGAAGTTTG |  |
| FTL1065-P2 | TCCTCATCGCAGAAGACACACCAA |  |
| FTL1134-P1 | GTTTCGATGATTGGGACTGGC |  |
| FTL1134-P2 | TGTGGATCTACCTCTTTCACAAAG |  |
| FTL1143-P1 | AGGAAGGTGGTTGTGGGACATTTG |  |
| FTL1143-P2 | GCTGGAAAGCCACTTTCTCTGGAA |  |
| FTL1262-P1 | TTTGCGCTGATTAAGACGCCGAAC |  |
| FTL1262-P2 | ACCAACCGACTTAGCAAACGGAGA |  |
| FTL1273-P1 | CAACAACAACGGTTTCTGAGGAGG |  |
| FTL1273-P2 | AGGTTTGGTGGAATAAATTGAAACT |  |
| FTL1307-P1 | CCAACAAGTCTTCCTTCTCCAGTG |  |
| FTL1307-P2 | ACCCATATTTCACTAGCCGAATCA |  |
| FTL1311-P1 | TGCTAATATGGCTCGACTCGGCTT |  |
| FTL1311-P2 | CACGTCGTGAATTCCAAACGAGCA |  |
| FTL1323-P1 | TCTGGTGTGTATATAAGTTGATTT |  |
| FTL1323-P2 | TGAAGAATACATGATAGAGAA |  |
| FTL1369-P1 | CGACTTAATCAACTAAGCCCAGTG |  |
| FTL1369-P2 | GGTCGATCCGTACAAACTTTAGAGAC |  |
| FTL1371-P1 | GGTTCACATTTGGATAACGAGTCGGC |  |
| FTL1371-P2 | TGCATAGACAAGACTCTGTTGAGA |  |
| FTL1376-P1 | CCCACTAATCTATTCATCTCACTTAAA |  |
| FTL1376-P2 | CCTTTCGGACCAGTACCTGAAACT |  |
| FTL1405-P1 | ACTCCACCAGTTACACACTAACCC |  |
| FTL1405-P2 | AAGTAAGCAGCGTCAACACATCGG |  |
| FTL1413-P1 | ATATGTAAGGCGCCGGCATTAGGT |  |
| FTL1413-P2 | AGTTGAGATGATCTGGCGTTTGCC |  |
| FTL1431-P1 | TCTGGTTCTTCTGGGTTGTTTG |  |
| FTL1431-P2 | AGGTCAAGAGTGCAAGATCTGG |  |
| FTL1470-P1 | GGTTATCACACTCGCAACTAATGGGC |  |
| FTL1470-P2 | ACGTGGCATACTAGGGTTTAGTGG |  |
| FTL1478-P1 | GTCAAGTTTCCCTACCTCTGAATGC |  |
| FTL1478-P2 | ACGCCTACCTCTTTGTCCATAAGAGT |  |
| FTL1500-P1 | CCCAAATCAGTAGCGCACACAACA |  |
| FTL1500-P2 | TCTTAGCCGAAGGGCAGACATCTT |  |
| FTL1506-P1 | CACGTCATTGCCAACGTCATTCGT |  |
| FTL1506-P2 | AGGAGGTTTAGGTTGCCGGAGAAT |  |
| FTL1518-P1 | TCATACTTGTCTCTTCCGGTCCGT |  |
| FTL1518-P2 | CATCCATGAAATGTGGGAAATTGGT |  |
| FTL1524-P1 | TCTGGTGACGATGACCAAAGGGTT |  |
| FTL1524-P2 | GCCTAGTCATAAGCAGTGAAACCTC |  |
| FTL1614-P1 | TGTTATGTTGACTACGCTTCGCCG |  |
| FTL1614-P2 | CGAAGTACTATCGTTCTCGGGTGC |  |
| FTL1618-P1 | GGTTGGTTAGCTAATGGTG |  |
| FTL1618-P2 | ACGTGTGCATAATCAAA |  |
| FTL1659-P1 | CCCATCAACTTAGAAAGTCTAATGAA |  |
| FTL1659-P2 | GGGTGGGCCTTATTGGATATGA |  |
| FTL1805-P1 | TGCTCTTCTTCTTCGGTTCGGGTT |  |
| FTL1805-P2 | ACGTTAACCGCATGAAGGTTGCTG |  |
| FTL1822-P1 | ACTTTAGAGTACTACTTGTTTAGA |  |
| FTL1822-P2 | TGACGTACACCACTAACTGA |  |
| FTL1997-P1 | GATGGTCGTAGTACCACACTCTCT |  |
| FTL1997-P2 | AGATTCCTTACGTTGAAGCTACC |  |
| FTL2015-P1 | GTGGGCTAGTATTGTGCTTACGGT |  |
| FTL2015-P2 | CGGGCTAAGCAACTCACAACGATT |  |
| FTL2066-P1 | ATGTTCGTGTCCACCAAGGTCAGT |  |
| FTL2066-P2 | AAATACAGGCGACACAAGCGGATG |  |
| FTL2133-P1 | GTGTATGTCCATAGGACTCGACACT |  |
| FTL2133-P2 | ACGCCTAAAGTAAGATTCTCACAC |  |
| FTL1997-P1 | GATGGTCGTAGTACCACACTCTCT |  |
| FTL1997-P2 | AGATTCCTTACGTTGAAGCTACC |  |
| FTL2180-P1 | GAATACGGTTTGTTTCCTGGAG |  |
| FTL2180-P2 | AATAGTCCACGCTTGTTCTCTCTG |  |
| FTL2201-P1 | GTAACGGGCTTTGAAATGGGCTGA |  |
| FTL2201-P2 | TCCCTAACTCTTTCTTCGTCTTTCCC |  |
| FTL2217-P1 | CCTACTCTTCCAAGTTCCAACCAACG |  |
| FTL2217-P2 | CCATCTGATGAGAGATAGAGCGTG |  |
| FTL2261-P1 | GGATGCACACGTCTATATACACAC |  |
| FTL2261-P2 | GGACTACTCCTTCTCCTCTGCAC |  |
| FTL2271-P1 | CCTACTCTTCCAAGTTCCAACCAACG |  |
| FTL2271-P2 | CCATCTGATGAGAGATAGAGGGTG |  |
| FTL2376-P1 | AAAGTAGAGTGCCACATGTAGACC |  |
| FTL2376-P2 | GTTGATTCCGGAGCATTGGTC |  |
| FTL2385-P1 | TTGTGTCTCCGTGTAGTTGACC |  |
| FTL2385-P2 | ATGTTCTCTAACTTCGTCGGTG |  |
| FTL2450-P1 | CGAACCACCGTATAAACAAACACC |  |
| FTL2450-P2 | CAGAGTGAAACTGAGTAAAGAGTC |  |
| FTL1456-P1 | AGAAATCATGAGCTGAGCTGT |  |
| FTL1456-P2 | CGACCTCAAGCTCCAAAGTTACAC |  |
| FTL2616-P1 | CATCCGAGTAATAAGACTAGTTCG |  |
| FTL2616-P2 | TGCCCTATTGAAGAAAGAGT |  |
| FTL2634-P1 | ACAAAGCCGTGCGAGTTCTAATG |  |
| FTL2634-P2 | AGCATCTGCGAGGTGAATTAACC |  |
| FTL2725-P1 | TGCATAGAAGGCATGAATCGATAG |  |
| FTL2725-P2 | GCATGTTTGCCAACCATATTCAAC |  |
| FTL2780-P1 | GTCGTTCCAAGCAACCCACTCC |  |
| FTL2780-P2 | GTTGCTCTCTGCTCCTGGTTCC |  |
| FTL2815-P1 | CAATCTTCAACATCGCCGTGTTAC |  |
| FTL2815-P2 | CGAGTGGTCACTACCTTGGTCACC |  |
| FTL2913-P1 | TTTAATGCTGGTGTACGAG |  |
| FTL2913-P2 | CAGCCTTAAACCCTGTAGTC |  |
| FTL2914-P1 | CGAACCTGCTATCTTTGCTCTTG |  |
| FTL2914-P2 | GCCCATTGGTTGATCTCATGTG |  |
| FTL3115-P1 | TCATGTAATGCGACGACTATGG |  |
| FTL3115-P2 | GACCGTTGATTTAGGGACACTG |  |
| FTL3263-P1 | GATTCAGACACAAGGACCGACC |  |
| FTL3263-P2 | ACTTTGAATTCCCACGACGCAG |  |
| FTL3269-P1 | TCCGAACCTGACCTGAATACCG |  |
| FTL3269-P2 | TTAACTTGACGAGCAACGGAAG |  |
| FTL3282-P1 | GGAATCTGCGGTGAGTTTGAAG |  |
| FTL3282-P2 | TGCGTAAGGATGGTCGTAATGTG |  |
| FTL3304-P1 | CGTACACGTAACGGCGACTATCC |  |
| FTL3304-P2 | CAGATCTCCCAAACCCTAACTTCC |  |
| FTL3379-P1 | CAGGCCACGTCATAAACTCA |  |
| FTL3379-P2 | AAAGAAGTTTCGCCCTCCAT |  |
| FTL3411-P1 | CAAACCGATTTGCTGTGAACTC |  |
| FTL3411-P2 | GGACGTGGTATGTATTAGTTAGCC |  |
| FTL424-P1 | CGGACTCTGTCTTCTCCACAAA |  |
| FTL424-P2 | TCTCAGTTAGTTGACTTTACAGC |  |
| FTL567-P1a | TGGTCGGCCCTAAATGTTTG |  |
| FTL567-P2a | ACCGACACAAGAATCTGTGGAACC |  |
| FTL567-P1b | ACGAAATGTGGGTAAATCTGACGC | also works with FTL567-P2a |
| FTL567-P2b | TATTGACCGCCAAGGAATTAGC | also works with FTL567-P1a |
| FTL800-P1 | GGAAGACCATTATAGTACGGAGCC |  |
| FTL800-P2 | GGTAGAGAATCCAAATCCGTATCAAC |  |
| FTL804-P1 | TGGTCCTTGGACAACTTCTCTGCT |  |
| FTL804-P2 | GGTTGTTCACTCGAGGTTTCGTCA |  |
| FTL965-P1 | CAGGATTCAGGACAAAGGATTTGG |  |
| FTL965-P2 | TCCTATGTACTTCCCTTGCCACTG |  |
| FTL992-P1 | CCATGTTCAGTTAACACAAGAAGAGT |  |
| FTL992-P2 | CTTCAAGAAGCGGTGCAGTCACAT |  |
| FTL993-P1 | AACTCATGATCGTTTGTTTACT |  |
| FTL993-P2 | GATTCGTGATGGCGACGATTA |  |
| FTL1963-P1 | ACTTACACGTGTGCCTTCTCATGC |  |
| FTL1963-P2 | AGCCAGTACTCGAATACGCCATGT |  |
| FTL1625-P1 | GCAGCTGCAAGAGTACCAAGGAAA |  |
| FTL1625-P2 | GCATTGAATGGAGCAGGTGGTGAT |  |
| FTL2504-P1 | CAACGGAGAAGCAAACTCAATACA |  |
| FTL2504-P2 | TGGAAGGATCAAGGATGGAACC |  |
| FTL-L1 | CAATTCGGCGTTAATTCAGTAC | used with P1/P2 or as 2° LMS-PCR primer 332 bp from left T-DNA border |
| FTL-L2 | CTATGTTACTAGATCGACCGG | alternate 1° LMS-PCR primer 370 bp from left T-DNA border |
| FTL-R1 | CCGACCCAGCTTTCTTGTAC | used with P1/P2 or as 1° LMS-PCR primer 500 bp from right T-DNA border |
| FTL-R2 | CTTATAGGGTTTCGCTCATGTG | alternate 2° LMS-PCR primer 317 bp from right T-DNA border |
| ADAPL-E1 | CTAATACGACTCACTATAGGGCTCGAGCGGCCGCCCGGGCAGGTG | LMS-PCR, long adapter for FTL lines |
| ADAPS-E1 | AATTCACCTGCCCGG/3AmMc7 | LMS-PCR, EcoR I adapter for FTL lines |
| ADAPS-blunt | 5Phos/CACCTGCCCGG/3AmMc7 | LMS-PCR, blunt adapter |
| ADAPS-HindIII | 5Phos/AGCTCACCTGCCCGG/3AmMc7 | LMS-PCR, Hind III adapter |
| ADAPS-AseI | 5Phos/TACACCTGCCCGG/3AmMc7 | LMS-PCR, AseI adapter |
| AP1 | GGATCCTAATACGACTCACTATAGGGC | LMS-PCR primer for 1° PCR |
| AP2 | TATAGGGCTCGAGCGGCCG | LMS-PCR primer for 2° PCR |
| PGWLat52LB-RB-WP1 | CCCAGAATCGATCATTCCTC | 1° LMS-PCR primer 1831bp from left T-DNA border |
| PGWLat52RB-RB-WP2 | ACGAATTCCTGCAGCCCG | 2° LMS-PCR primer 1786bp from left T-DNA border |
| ADAPS-blunt | 5Phos/CACCTGCCCGG/3AmMc7 | LMS-PCR, blunt adapter |
| ADAPS-HindIII | 5Phos/AGCTCACCTGCCCGG/3AmMc7 | LMS-PCR, Hind III adapter |
| ADAPS-AseI | 5Phos/TACACCTGCCCGG/3AmMc7 | LMS-PCR, AseI adapter |
| Lat52 F | CAGAAGGTATTGAGGAATGATCG | amplifies probes for Southern blot |
| Lat52 R | TGCTCCTTCTCTTTGTGTGTGTC | amplifies probes for Southern blot |
